# Pollen Tube Growth and Self-Compatibility in Almond

**DOI:** 10.3390/plants2010050

**Published:** 2013-02-04

**Authors:** Rafel Socias i Company, Ossama Kodad, Àngel Fernández i Martí, José M. Alonso

**Affiliations:** Unidad de Fruticultura, Centro de Investigación y Tecnología Agroalimentaria de Aragón (CITA), Av. Montañana 930, 50059 Zaragoza, Spain; E-Mails: okodad@aragon.es (O.K.); afernandez@pctad.com (A.F.iM.); jmalonsos@aragon.es (J.M.A.)

**Keywords:** almond, *Prunus amygdalus* Batsch, pollen tube growth, self-compatibility

## Abstract

Although pollen tube growth has been an important criterion for self-compatibility evaluation in almond, there is not a clear-cut separation between positive and negative growth of pollen tubes in the different genotypes. The examination of pollen tube growth after selfing almond seedlings has allowed establishing different levels of compatibility, but not a clear-cut separation between self-compatible (SC) and self-incompatible (SI) genotypes, related to the presence of pseudo-self-compatibility in almond. Consequently, a relationship between pollen tube growth and self-compatibility in almond may be established for evaluating the seedlings in breeding programs.

## 1. Introduction

At present, most almond (*Prunus amygdalus* Batsch) breeding programs aim at developing self-compatible (SC) cultivars to overcome the problems related to cross-pollination of this mostly self-incompatible (SI) species [1]. Consequently, self-compatibility (SCy) is a primary trait to be considered during evaluation of seedlings of breeding programs [2]. Effective SCy implies, firstly, pollen tube growth (PTG) after self-pollination similar to that after cross-pollination with cross-compatible pollen [3]. Secondly, this good PTG after self-pollination should result in similar fruit sets, which may not always be the case [4]. And thirdly, these fruit sets must reach the level of a commercial crop [5]. From a horticultural point of view there is a fourth requirement that these fruit sets must be obtained by autogamy, the ability of a genetically self-compatible cultivar to pollinate itself in the absence of insects [6].

As the presence of the *S_f_* allele was supposed to confer SCy to almond [7], the detection of SC seedlings has been also undertaken by the identification of this allele by PCR analysis in the offspring of crosses [8,9]. However, this information is only genetic, not horticultural, because the presence of the *S_f_* allele is not always related to SCy [10,11,12]. In addition, there is not a clear-cut separation between SC and SI genotypes, showing that a quantitative effect may condition the expression of SCy [13], related to the presence of pseudo-SCy (PSCy) in almond [2]. Several approaches have been used to assess the level of SCy in almond, with every method showing advantages and limitations [14]. However, the final evaluation of SCy of a cultivar or selection is its productivity under field conditions, *i.e*., with solid blocks of one clone isolated from any other almond clone and even in the absence of pollinating insects, taking into account that this production must attain a commercial level.

Since the first observations of PTG in almond [3], the experience of nearly 40 years has shown that there is not a clear-cut separation of genotypes according to PTG after selfing. All flowers of the same genotype may not have the same PTG pattern, which may also show differences according to the year. This continuous variability has been also observed for fruit set [2], but when compatible cross-pollinations are studied, PTG reflects this compatibility by the presence of pollen tubes in the style base of all pistils [3]. Accordingly, our objective was to establish a PTG ratio which could be related to the real level of SCy of a genotype.

## 2. Results and Discussion

PTG has been considered a clear indication of the compatibility of any pollination as it is independent of the environment where the study is done, on the tree in the field, on branches taken from the tree and brought to the lab or in trays as described in this study [15], as in all cases the results have been unequivocal. However, the studies conducted in the field are subject to unpredictable weather conditions such as frosts. Frosts may destroy the pistils, but this is not the case of the pollen tubes, that only suffer growth arrest at low temperatures [16]. The environmental conditions not only affect PTG in the field, but also the operations of emasculation and pollination. These operations are carried out for comparing self- and cross-pollination in the open air. Temperatures are usually very low at almond blooming time in many almond growing regions and, if winds are blowing, much attention must be paid to conduct these operations. Thus, PTG determination in the field is mostly restricted for elucidating doubtful results observed from laboratory pollinations.

The studies conducted in the field show the most reliable response since they reflect the natural conditions of the pollination. Field studies also offer more reliable results as pollen tubes from these pistils are identified much more easily [3]. However, the weather contingencies affecting these studies must be avoided. This may be accomplished by taking single flower buds as reported in this study, additionally saving space. Furthermore, the trays with the pollinated flowers can be kept in chambers to control temperature. Higher temperatures than usual increase the speed of compatible PTG but aggravate the symptoms of pollen incompatibility [3].

**Table 1:** Seedling classification in SC indices of the offspring from the almond cross G-4-3 × “Marcona” according to their PTG score.

SC index	SC classification	PTG score	Genotypes
**1**	Fully SI	<50.1	P-7-68, P-7-49, P-7-53, P-7-60, P-7-76, P-7-82, P-7-87,P-7-92, P-8-14, P-8-27, P-8-32, P-8-69, P-8-70, P-8-72,P-8-86, P-8-87, P-8-98
**2**	SI	50.1–60	P-7-51, P-8-83, P-7-38, P-7-86, P-8-57, P-8-77, P-8-99,P-8-90, P-8-25, P-8-68, P-7-48, P-8-9, P-8-97, P-7-67,P-7-42, P-8-19, P-7-50, P-8-48, P-7-95, P-8-78, P-7-62,P-8-74, P-7-91
**3**	SI doubtful	60.1–70	P-7-61, P-7-69, P-8-89, P-8-62, P-8-34, P-8-43, P-8-23,P-8-16, P-8-91, P-8-81, P-8-18, P-7-74, P-7-45, P-7-43,P-8-28, P-7-36, P-8-45, P-7-41, P-8-94, P-7-81, P-8-8,P-7-37, P-7-64, P-8-39, P-8-20, P-7-75, P-8-6
**4**	Doubtful	70.1–80	P-8-13, P-7-90, P-8-61, P-8-17, P-8-71, P-8-40, P-7-96,P-7-98, P-7-52, P-8-11, P-7-44, P-8-73, P-7-89, P-8-76,P-8-5, P-7-97, P-8-24, P-8-51, P-8-56, P-7-73, P-8-96,P-8-49, P-7-65, P-8-26, P-7-80, P-8-42, P-7-59, P-8-59,P-8-82, P-8-80, P-8-84, P-7-79, P-8-35, P-8-4, P-8-55,P-8-92, P-8-38, P-7-70, P-8-22, P-8-47, P-8-12, P-7-57,P-8-15
**5**	SC doubtful	80.1–90	P-7-78, P-8-54, P-8-46, P-7-85, P-8-7, P-8-95, P-8-2,P-8-41, P-8-88, P-8-30, P-8-66, P-8-67, P-8-31, P-8-75,P-7-40, P-8-93, P-8-50, P-7-94, P-7-84, P-7-63
**6**	SC	90.1–99.9	P-8-58, P-8-85, P-8-53, P-8-10, P-8-29, P-8-52, P-8-65,P-8-60, P-7-39, P-7-56, P-7-83, P-8-1, P-8-79, P-7-35,P-8-63, P-7-47, P-7-71, P-8-36, P-8-3, P-7-46, P-7-77,P-8-44, P-8-64
**7**	Fully SC	100	P-7-54, P-7-55, P-7-58, P-7-66, P-7-72, P-7-88, P-7-93,P-7-99, P-8-21, P-8-33

Our observations of PTG following self-pollination in the laboratory for all individuals of the breeding progeny allowed rating each genotype according to the score established for PTG. After this rating, only 10 out of the 163 seedlings of the population studied could be rated as fully SC because all pistils showed pollen tubes at their style base, with a score of 100. Conversely, 17 genotypes could be rated as fully SI because any pistil did not show pollen tubes growing down the middle third of the style. A single genotype (P-7-68) had a score lower than 50 because some pistils only showed PTG just under the stigma, thus lowering the mean ratio. All intermediate scores were obtained, in a continuous variation which enabled classifying the genotypes according to their SC index (Table 1). SC index 1 was applied to genotypes with a PTG score lower than 50.1, indicating a full SI. SC index 2 was applied to genotypes scored from 50.1 to 60, also considered fully SI, despite that some pistils could have pollen tubes growing a little more down, near the style base in order to increase the mean score. Similarly, SC index 3 was applied to genotypes with scores from 60.1 to 70 and classified as SI, but with some doubts on their level of SC/SI. SC index 4 was for scores from 70.1 to 80 of genotypes classified as doubtful. SC index 5, applied to scores from 80.1 to 90, defined genotypes with some level of SC, but also with some doubts on their level of SC/SI. SC index 6 was applied to genotypes considered SC, with scores from 90.1 to 100, indicating that some pistils did not shown pollen tubes at their style base. This may be expected even in fully SC genotypes, as flower manipulation during preparation could damage the pistil and reduce PTG. Finally, SC index 7, with a score of 100, was applied to fully SC genotypes.

**Figure 1:**
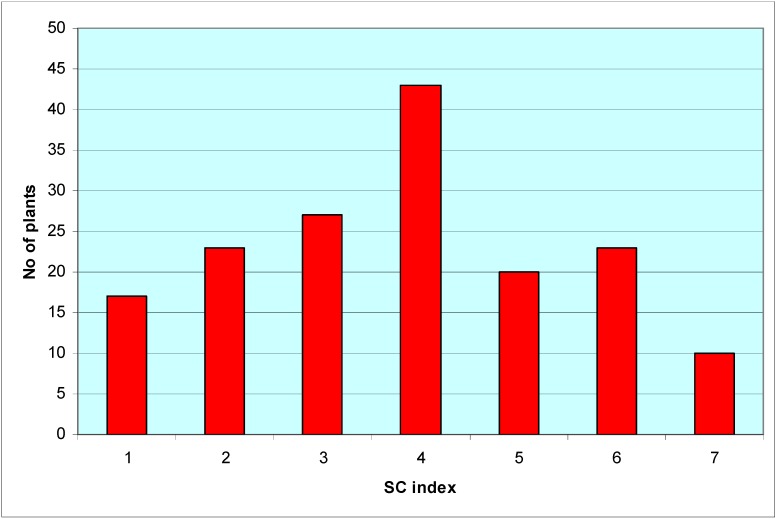
Distribution of the offspring of the almond cross G-4-3 × “Marcona” according to their self-compatibility index (1: Fully self-incompatible; 7: Fully self-compatible; 2–6: intermediate; see Table 1).

When the number of individuals in each SC index was considered, they approached a normal distribution (Figure 1), with a χ^2^ of 8.51 with 4 df, thus showing a quantitative expression of PTG in this population. In addition, the mean value of this population (74.1) was very close to the median (73.5).

PTG has often been associated with fruit setting following artificial pollinations, giving similar results [4,17,18]. However, our results show that SC in this population cannot be attributed to a qualitative effect, distinguishing SC from SI genotypes, but to a quantitative one. Although inbreeding depression may affect the expression of SCy by PTG [19], this could not be the case in this population as the parents are not related [20].

Socias i Company [2] had already suggested that almond is a SI species with a genetic background of PSCy as indicated by the small self set observed in some cultivars. Over this background, only one *S_f_* allele could break the SI system, but probably interacting with this background of PSCy, as shown by the present results of PTG. Since the first work by Almeida [21], all range of fruit sets has been described when self-pollinating almond cultivars [2]. Additionally, Fernández i Martí *et al*. [13] identified two QTLs affecting almond SC, sustaining the quantitative nature of SC in almond. From the agronomical point of view fruit set has been considered the main evaluation criterion for SC selection in almond [22]. However, the joint effect of the *S_f_* allele and other genes, such as these QTLs recently identified, might explain this wide range of both PTG and fruit set, independently of the changing year effect on these sets [23], as it has also been described in Japanese pear [24], suggesting that pollen factors unrelated to the *S* locus affect PTG and fruit set.

## 3. Experimental Section

The cross G-4-3 × “Marcona” from the CITA almond breeding program was selected for this study. It includes 163 seedlings and was designed between a late-blooming SC selection from the same breeding program (G-4-3, genotype *S_11_S_f_*, from the cross “Felisia” × “Bertina”) and a traditional Spanish early-blooming and SI cultivar (“Marcona”, genotype *S_11_S_12_*). The study was carried out during five consecutive years depending on the availability of flowers on the young seedlings. Each genotype was studied two years if the results of both years were concordant, but some were examined along the five years of study in order to confirm their compatibility behavior.

Flowers at stage D [25] were collected from each seedling and taken in plastic bags at the laboratory. Twenty flowers per seedling were emasculated and placed in trays with tap water, with the peduncles passing through the holes of a plastic mesh floating on the water over several pieces of wood. Anthers were extracted from the same flowers and left to dry on paper trays for 48 h, after what the pistils were self-pollinated. After pollination the pistil trays were placed in constant temperature chambers at 22 °C and the pistils were collected from the trays 96 h after pollination to allow pollen tubes reaching the style base [3]. The pistils were autoclaved in a 5% solution of Na_2_SO_3_ for 12 min at 1.2 kg cm^−2^. The samples were maintained at 2–4 °C until observation.

If after two years of observation the results were not conclusive, PTG was additionally observed in the field by emasculating a minimum of 20 flowers at stage D on the original plant and self-pollinating the pistils two days later. Pistils were collected 10 days after pollination to allow pollen tubes reaching the style base [16] and treated similarly to those collected from the trays in the laboratory.

For PTG observation the pistils were prepared according to the method of Socias i Company [26], dissecting the outer part of the pistils and leaving only the transmitting tissue trough which pollen tubes grow. This growth was assessed by observation in a Leitz Ortholux II microscope with UV illumination of a mercury lamp Osram HBO 200 W/4, by fluorescence of the callose deposits of the pollen tubes by aniline blue staining after squashing the pistils [27]. The pistils were rated according to the level where pollen tubes were observed as defined in Table 2. Finally, each genotype was classified according to the average rate of all the pistils observed, pooling the data of all years of observation in order to obtain the SC index for the genotype.

**Table 2:** Rating of the almond pistils according to the level of pollen tube growth.

Level of PTG	Rating
Pollen tubes at the style base	100
Pollen tubes near the style base with no signs of incompatibility, thus suggesting that pollen tubes could reach the ovary	95
Pollen tubes near the style base with signs of incompatibility, thus suggesting that pollen tubes could not reach the ovary	90
Pollen tubes reaching the middle third of the pistil	50
Pollen tubes just penetrating the style, but not growing down	10
Germination of pollen grains but pollen tubes not penetrating the style	5
Pollen grains on the stigma but no pollen germination	0
No pollen grains on the stigma	Not included in the analysis

## 4. Conclusions

PTG showed a continuous variation in the almond population studied. Thus, for SCy evaluation, an SC index may be established in order to rate the genotypes according to the PTG assessed in their pistils after self-pollination. Consequently, only genotypes showing SC indices of 6 (SC) and 7 (fully SC) could be rated as SC and be further considered for evaluation in an almond breeding program aimed at the release of commercial SC cultivars.
